# Trauma Exposure, Insomnia, and Fatigue: A Cross‐Sectional Study of the Pathways to Burnout Among South African First Responders

**DOI:** 10.1002/hsr2.71204

**Published:** 2025-08-28

**Authors:** Anita Padmanabhanunni, Tyrone B. Pretorius

**Affiliations:** ^1^ Department of Psychology University of the Western Cape Cape Town South Africa

**Keywords:** burnout, fatigue, first responders, Insomnia, South Africa, trauma exposure

## Abstract

**Background and Aims:**

First responders are disproportionately vulnerable to the development of insomnia, fatigue and burnout, due to chronic exposure to trauma inherent in their occupational roles. This study examined the mediating roles of insomnia and fatigue in the relationship between trauma exposure and burnout among South African first responders.

**Methods:**

Participants included police officers (*n* = 309) and paramedics (*n* = 120) in the Western Cape province of South Africa. They completed an online survey comprising the Life Events Checklist, the Insomnia Severity Index, the Chalder Fatigue Questionnaire, and the Maslach Burnout Inventory. Mediation analyses were conducted using the Hayes PROCESS macro in SPSS Version 30.

**Results:**

Mediation analysis provided evidence of both parallel as well as serial mediation roles for insomnia and fatigue in the relationship between trauma exposure and burnout. Insomnia fully mediated the relationships between trauma exposure and emotional exhaustion as well as personal accomplishment and partially mediated the relationship with depersonalization. Fatigue fully mediated the relationships between trauma exposure and emotional exhaustion and depersonalization but did not mediate the relationship with personal accomplishment. Serial mediation analysis indicated that insomnia and fatigue, in sequence, fully mediated the effects of trauma exposure on emotional exhaustion and depersonalization.

**Conclusions:**

Insomnia and fatigue are critical pathways linking trauma exposure to burnout among South African first responders. Early identification and treatment of sleep disturbances may be crucial for preventing burnout and enhancing resilience in this population.

## 1 Introduction

First responders are essential to crisis management, providing rapid assessment and intervention in high‐pressure environments. They are often the first to arrive at the scene of emergencies, natural disasters, or accidents, delivering immediate support. This group includes paramedics, police officers, firefighters, and emergency medical technicians, who manage life‐threatening situations and undertake rescue operations [1]. The chronic exposure to potentially traumatic events in these environments significantly increases the risk of developing posttraumatic stress disorder (PTSD). PTSD is characterized by symptoms such as intrusive re‐experiencing (e.g., flashbacks, nightmares), avoidance of trauma reminders, negative alterations in mood and cognition (e.g., guilt, detachment), and heightened arousal and reactivity [2]. These symptoms impair occupational functioning and reduce quality of life, compounding the psychological burden faced by individuals working in high‐risk environments [3].

The constant demand for 24‐h emergency services necessitates irregular work hours and shift work, disrupting circadian rhythms, impairing sleep, and contributing to insomnia and burnout [4, 5]. Insomnia, characterized by difficulties initiating or maintaining sleep despite adequate opportunity, is highly prevalent among first responders. It adversely affects daytime functioning and quality of life [2, 6, 7]. Insomnia is associated with physical (e.g., sense of tiredness and loss of energy) and cognitive fatigue, defined as a decrease in cognitive efficiency particularly in the context of mentally demanding tasks [8]. It is a risk factor for cardiovascular disease, neurological disorders (e.g., Parkinson's and Alzheimer's disease) and co‐occurs with other mental health disorders, notably depression and anxiety [9, 10]

Recent research findings suggest that a substantial proportion of first responders, particularly those with insomnia, exhibit heightened PTSD symptoms [3, 11]. This has been attributed to fear of sleep [11]. Individuals with PTSD frequently experience distressing nightmares and intrusive memories during sleep, leading to the development of anticipatory anxiety about sleeping [12]. This fear can manifest as a reluctance to go to bed, prolonged sleep onset latency, and frequent nocturnal awakenings. Over time, fear of sleep becomes a learned response, reinforcing sleep disturbances and contributing to chronic insomnia [11]. The resulting sleep deprivation further intensifies PTSD symptoms, including emotional dysregulation, heightened arousal, and impaired cognitive functioning, creating a cycle that is difficult to interrupt [11, 12, 13].

Burnout occurs as a consequence of consistent and prolonged exposure to stressors. It is a multidimensional construct consisting of emotional exhaustion, depersonalization (e.g., sense of cynicism, loss of enthusiasm and detachment towards one's work) and reduced sense of personal accomplishment [14]. Existing research highlights a complex, bidirectional relationship between insomnia and burnout, with each condition capable of exacerbating the other over time [7, 15, 16]. Insomnia can lead to persistent physical exhaustion, impaired cognitive functioning, and heightened emotional reactivity. These symptoms, in turn, can contribute to the development or worsening of burnout, particularly by diminishing an individual's capacity to manage occupational stressors effectively [15]. Conversely, burnout can perpetuate sleep disturbances by maintaining a state of chronic psychological arousal and rumination, making it increasingly difficult to achieve restorative sleep [17]. The consistent depletion of physical and emotional resources, combined with escalating levels of stress, creates a self‐reinforcing cycle that can significantly impair both mental health and occupational functioning [7, 18, 19].

The current study is grounded in the Conservation of Resources (COR) theory [20] which posits that individuals strive to obtain, retain, and protect resources that are essential for their well‐being. According to COR theory, psychological stress occurs when there is a threat of resource loss, an actual loss of resources, or a failure to gain resources following investment. In the context of trauma‐exposed first responders, chronic exposure to potentially traumatic events depletes critical personal resources, including emotional energy and cognitive functioning. Insomnia and fatigue represent key indicators of such resource depletion, undermining the ability to manage occupational demands effectively.

Accordingly, this study examines the potential roles of insomnia and fatigue in mediating the relationship between trauma exposure and burnout among South African first responders.

## 2 Methods

### 2.1 Participants and Procedure

Participants were 429 first responders, comprising police officers (*n* = 309) and paramedics (*n* = 120) from the Western Cape province of South Africa. A post hoc power analysis was conducted using the “medserial” function in the pwr2ppl package (Aberson, 2019) in R (R Development Core Team, 2020). Based on the coefficients obtained in the current study and a sample size of 429, the results indicated that the study achieved adequate statistical power, which is generally considered sufficient when exceeding 80% [21]. Specifically, in the serial mediation model (see Data Analysis), which included two mediators, the power estimates were 87.7% for the first mediator, 83% for the second mediator, and 87.7% for the serial mediation path.

An electronic version of the survey instruments was created, and with the permission of Facebook group administrators for first responders, an invitation to participate was posted online alongside the survey link.

Due to South Africa's privacy laws restricting access to databases of first responders, random sampling was not feasible. In addition to online recruitment, permission from the Western Cape Department of Health (reference: WC_202307_041, 15 September 2023) and the South African Police Services (reference: 3/34/2, 27 June 2023) enabled research assistants to recruit participants at hospitals and police stations.

Table 1 presents a comprehensive demographic profile of the study participants. The sample consisted of 429 first responders, the majority of whom were men (55%; *n* = 236), with women making up 45% (*n* = 193) of the sample. In terms of job roles, most participants were police officers (72%; *n* = 309), while paramedics accounted for 28% (*n* = 120) of the sample.

**Table 1:** Description of the sample of first responders.

Variable	Category	*n*/Mean	%/SD
Gender	Women	193	45.00%
	Men	236	55.00%
Job title	Police Officers	309	72.00%
	Paramedic	120	28.00%
Area of residence	Urban	396	92.30%
	Peri‐Urban	11	2.60%
	Rural	22	5.10%
Education	Less than Grade 12	5	1.20%
	Grade 12 certificate	211	49.20%
	Post‐Matric Diploma	145	33.80%
	Undergraduate Degree	41	9.60%
	Postgraduate Degree	27	6.30%
Relationship status	Single	151	35.20%
	Married	221	51.50%
	Divorced or Separated	57	13.30%
Age		39.00	9.93
Length of service		13.24	9.65
Number of dependents		2.36	1.86

The mean age of participants was 39 years (SD = 9.93), reflecting a mid‐career cohort. Participants reported an average of 13.24 years of professional experience (SD = 9.65) in their roles as first responders. On average, participants had approximately two dependents (M = 2.36; SD = 1.86).

Regarding residential location, the majority resided in urban areas (92.3%; *n* = 396), with smaller proportions residing in peri‐urban (2.6%; *n* = 11) and rural areas (5.1%; *n* = 22). Educational attainment varied, with nearly half of the participants having completed Grade 12 (49.2%; *n* = 211). Around one‐third held a post‐matric diploma (33.8%; *n* = 145), and smaller proportions held undergraduate (9.6%; *n* = 41) or postgraduate degrees (6.3%; *n* = 27). A minority reported educational attainment below Grade 12 (1.2%; *n* = 5).

With respect to relationship status, approximately half of the sample was married (51.5%; *n* = 221), while over a third were single (35.2%; *n* = 151), and 13.3% (*n* = 57) indicated being divorced or separated.

### 2.2 Measures

Participants completed a demographic questionnaire and four standardized measures:

- Life Events Checklist for DSM‐5 (LEC‐5 [22]:): A 17‐item measure assessing lifetime exposure to potentially traumatic events. Sixteen items assess specific event types e.g., natural disaster, transportation accident), and one open‐ended item captures any other distressing experience. Participants respond to the items of the LEC on a six‐point scale indicating whether an event had happened to them, whether they witnessed it, whether they learned about it, whether it's part of their job, whether they are unsure, and whether it does not apply. Events were weighted according to proximity: direct exposure (3), witnessed (2), and learned about the event (1), as recommended by Weis et al. [23]. Previous studies reported satisfactory reliability (α = 0.83–0.87: [23, 24]).
- The Insomnia Severity Index (ISI: [25]): A 7‐item screening tool for insomnia that assesses the nature and symptoms of the respondents’ sleep problems using a 5‐point Likert‐type scale. An example of an item of the ISI is “how satisfied are you with your current sleep pattern.” In the original validation study, the authors of the scale reported an estimate of internal consistency of 0.74 for ISI scores and demonstrated that the ISI was sensitive to changes in sleep difficulties resulting from treatment. Although the ISI has previously been used in South Africa (e.g. [26, 27]), we could find no validation study or any reported estimate of reliability of the ISI scores when used with South African samples.
- The Chalder Fatigue Scale (CFQ: [28]): An 11‐item measure that assesses the severity of mental and physical fatigue on a 4‐point scale ranging from *less than usual* (0) *to much more than usual* (3). An example of an item of the CFQ is “do you lack energy.” In the development study, Chalder and colleagues (1993) provided estimates of internal consistency for CFQ scores that ranged between 0.88 and 0.90. A separate study cited correlations between the CFQ and five other measures of fatigue as evidence of convergent validity [29]. In South Africa only one study had previously used the CFQ and reported an estimate of internal consistency for CFQ scores of 0.83 [30].
- The Maslach Burnout Inventory (MBI: [31]): A 22‐item measure of three dimensions of burnout: emotional exhaustion, depersonalization, and personal accomplishment. Items are assessed on a 7‐point scale ranging from *never* (0) to *every day* (6). The emotional exhaustion scale (9 items) assesses feelings of being overextended in the work context and emotionally depleted. An example item of the emotional exhaustion dimension is “I feel used up at the end of the work day.” The depersonalization scale (5 items) assesses feelings of cynicism and callousness towards recipients of services. An example item of the depersonalization dimension is ”I have become more callous or uncaring towards people since I took this job.” The personal accomplishment scale measures feelings of competence and a sense of accomplishment in one's work. An example item of the personal accomplishment dimension is “I feel I am positively influencing other people's lives through my work.” Higher scores on emotional exhaustion and depersonalization and lower scores on personal accomplishment reflect greater experienced burnout. The authors reported reliability coefficients for the subscale scores that ranged between 0.77 and 0.84 and also provided evidence for convergent and discriminant validity [31]. The MBI has previously been used in South Africa, for example with university educators [32] and schoolteachers [33] and estimates of internal consistency for the subscale scores in the two studies ranged between.71 to 0.89 and 0.84 to 0.94, respectively.

### 2.3 Ethics

Ethical approval was granted by the Humanities and Social Sciences Research Ethics Committee at the University of the Western Cape (reference: HS23/2/4, May 23, 2023). The study adhered to the Declaration of Helsinki guidelines. Participants provided informed consent electronically before participation, and no incentives were offered.

### 2.4 Data Analysis

All statistical analyses were conducted using IBM SPSS Version 30. There were no missing data, as the survey design required completion of all items before proceeding to subsequent pages.

Descriptive statistics (means, standard deviations), distribution indices (skewness, kurtosis), internal consistency estimates (Cronbach's α and McDonald's ω), and Pearson correlations among variables were calculated. Skewness and kurtosis between −2 and +2 were considered indicative of approximate normality [34].

Mediation analyses were conducted using Hayes’ PROCESS macro (Model 6) to examine the parallel and serial mediating roles of insomnia and fatigue in the relationship between trauma exposure and burnout. The significance of indirect effects was evaluated using 95% confidence intervals.

All statistical tests were two‐tailed and level of statistical significance was set at.05.

## 3 Results

The descriptive statistics, distribution indices (skewness and kurtosis), internal consistency estimates (α and ω), and Pearson correlations among the study variables are reported in Table 2.

**Table 2:** Descriptive statistics, distribution indices, correlations, and reliabilities.

Variable/Scale	1	2	3	4	5	6
1. Trauma exposure	—					
2. Insomnia	0.15*	—				
3. Fatigue	0.20**	0.61**	—			
4. Emotional Exhaustion	0.16*	0.55**	0.57**	—		
5. Depersonalization	0.16**	0.47**	0.50**	0.80**	—	
6. Personal Accomplishment	0.01	0.20**	0.18**	0.39**	0.35**	—
Mean	23.8	10.9	14.4	24.9	12.5	29.5
SD	9.3	5.8	6.8	12.7	7.9	9.5
Skewness	−0.58	0.18	0.12	−0.02	0.10	−0.50
Kurtosis	0.49	−0.72	−0.25	−0.79	−1.06	0.01
Alpha	0.88	0.87	0.89	0.90	0.84	0.79
Omega	0.88	0.87	0.89	0.90	0.84	0.78

* *p* < 0.01

** *p* < 0.001.

Table 2 indicates that all variables demonstrated acceptable normality (skewness and kurtosis between −2 and +2). Reliability coefficients (α and ω) ranged between 0.78 and 0.90, indicating good internal consistency.

Trauma exposure was significantly positively associated with insomnia (*r* = 0.15, *p* = 0.002), fatigue (*r* = 0.20, *p* = 0.001), emotional exhaustion (*r* = 0.16, *p* = 0.001), and depersonalization (*r* = 0.16, *p* < 0.001), but not with personal accomplishment (*r* = 0.01, *p* = 0.94). In all instances it was a small effect size. The positive correlations indicate that higher levels of trauma exposure were associated with higher levels of insomnia, fatigue, emotional exhaustion and depersonalization.

Insomnia was significantly positively associated with all the indices of burnout (emotional exhaustion: (*r* = 0.55, *p* < 0.001, large effect size; depersonalization: *r* = 0.47, *p* < 0.001, medium effect size; personal accomplishment: *r* = 0.20, *p* < 0.001, small effect size). Similarly, fatigue was significantly positively associated with the indices of burnout (emotional exhaustion: (*r* = 0.57, *p* < 0.001, large effect size; depersonalization: *r* = 0.50, *p* < 0.001, large effect size; personal accomplishment: *r* = 0.18, *p* < 0.001, small effect size). Thus, higher levels of insomnia and fatigue, respectively, were associated with higher levels of burnout. The relationship between insomnia and fatigue was substantial and positive (*r* = 0.61, *p* < 0.001, large effect size).

The results of the mediation analysis are reported in Table 3 in terms of the direct effects of trauma exposure, insomnia, and fatigue on the indices of burnout, as well as the indirect effects of trauma exposure on burnout through insomnia and fatigue.

**Table 3:** Results of the mediation analysis with trauma exposure as predictor, insomnia and fatigue as serial mediators and burnout as dependent variable.

Effect	B	SE	95% CI	β	*p*
Direct effects					
Trauma exposure → EE	0.05	0.05	[−0.06, 0.15]	0.03	0.378
Trauma exposure → DP	0.05	0.04	[−0.02, 0.12]	0.06	0.166
Trauma exposure → PA	−0.04	0.05	[−0.14, 0.06]	−0.04	0.431
Insomnia → EE	0.69	0.11	[0.48, 0.89]	0.31	< 0.001
Insomnia → DP	0.36	.07	0.23, 0.50	0.27	< 0.001
Insomnia → PA	0.23	0.10	[0.03, 0.42]	0.14	0.022
Fatigue → EE	0.69	0.09	[0.52, 0.87]	0.37	< 0.001
Fatigue → DP	0.38	0.07	[0.27, 0.49]	0.32	< 0.001
Fatigue → PA	0.14	0.08	[−0.02, 0.31]	0.10	0.090
Indirect effects					
Trauma exposure → Insomnia → EE	0.07	0.02	[0.02, 0.12]	0.05	—
Trauma exposure → Insomnia → DP	0.03	0.01	[0.01, 0.07]	0.04	—
Trauma exposure → Insomnia → PA	0.02	0.01	[0.00, 0.05]	0.02	—
Trauma exposure → Fatigue → EE	0.06	0.03	[0.01, 0.11]	0.04	—
Trauma exposure → Fatigue → DP	0.03	0.01	[0.01, 0.06]	0.04	—
Trauma exposure → Fatigue → PA	0.01	0.01	[−0.00, 0.04]	0.01	—
Trauma exposure → Insomnia → Fatigue → EE	0.05	0.02	[0.01, 0.08]	0.03	—
Trauma exposure → Insomnia → Fatigue → DP	0.03	0.01	[0.01, 0.05]	0.04	—
Trauma exposure → Insomnia → Fatigue → PA	0.01	0.01	[−0.00, 0.03]	0.01	—

*Note:* DP = Depersonalization, EE = Emotional Exhaustion, PA = Personal Accomplishment.

Table 3 indicates that:

- In the presence of the mediators, trauma exposure had no direct effects on emotional exhaustion (β = 0.03, *p* = 0.378), depersonalization (β = 0.06, *p* = 0.166), and personal accomplishment (β = −0.04, *p* = 0.431).
- Insomnia had significant direct effects on emotional exhaustion (β = 0.31, *p* < 0.001), depersonalization (β = 0.27, *p* < 0.001), and personal accomplishment (β = 0.14, *p* = 0.022).
- Fatigue only had significant direct effects on emotional exhaustion (β = 0.37, *p* < 0.001) and depersonalization (β = 0.32, *p* < 0.001), but not on personal accomplishment (β = 0.10, *p* = 0.090).
- Insomnia mediated the relationships between trauma exposure and emotional exhaustion (β = 0.05, 95%CI [0.02, 0.12]), depersonalization (β = 0.04, 95%CI [0.01, 0.07]) and personal accomplishment (β = 0.02, 95%CI [0.00, 0.05]). A separate mediation analysis that included only insomnia as the mediator, showed that when considered on its own trauma exposure were significantly associated with emotional exhaustion (β = 0.15, *p* = 0.002) and personal accomplishment (β = 0.15, *p* = 0.002). However, in the presence of the mediator these associations were nonsignificant (emotional exhaustion: β = 0.08, *p* = 0.06; personal accomplishment β = −0.03, *p* = 0.580). The nonsignificant associations in the presence of insomnia would be indicative of full mediation, i.e. that the relationship between trauma exposure and emotional exhaustion and personal accomplishment are fully explained by insomnia. In the case of the relationship between trauma exposure and depersonalization, the association was still significant (β = 0.09, *p* = 0.029) in the presence of insomnia. This would indicate that insomnia only partially mediated the relationship between trauma exposure and depersonalization.
- Fatigue mediated the relationships between trauma exposure and emotional exhaustion (β = 0.04, 95%CI [0.01, 0.11]) as well as depersonalization (β = 0.04, 95%CI [0.01, 0.06]) but not between trauma exposure and personal accomplishment (β = 0.01, 95%CI [ − 0.00, 0.04]). A separate mediation analysis with only fatigue as the mediator demonstrated that this was full mediation as the relationships between trauma exposure and emotional exhaustion (β = 0.04, *p* = 0.286) as well as depersonalization (β = 0.07, *p* = 0.127) were nonsignificant in the presence of fatigue.
- Insomnia and fatigue, in sequence, mediated the relationships between trauma exposure and emotional exhaustion (β = 0.03, 95%CI [0.01, 0.08]) as well as depersonalization (β = 0.04, 95%CI [0.01, 0.05]) but not the association between trauma exposure and personal accomplishment (β = 0.01, 95%CI [ − 0.00, 0.03]). In both instances it was full mediation as the relationships between trauma exposure and emotional exhaustion (β = 0.03, *p* = 0.378) and depersonalization (β = 0.05, *p* = 0.166), respectively, were nonsignificant in the presence of insomnia and fatigue, in sequence.

**Figure 1:**
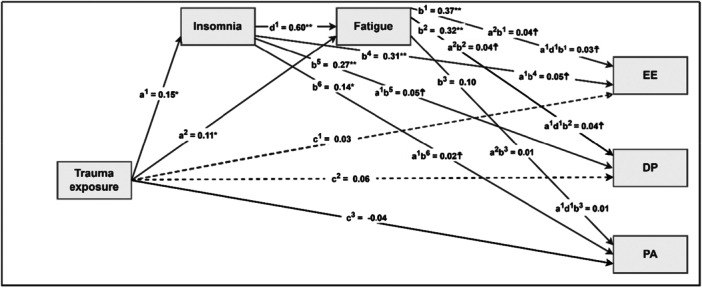
Visual Representation of the Parallel and Serial Mediating Roles of Insomnia and Fatigue. *Note:* DP = depersonalization, EE = emotional exhaustion, PA = personal accomplishment, c^1^–c^3^ = direct effects of predictor on dependent variables; a^1^–a^2^ = direct effects of predictor on mediators; d^1^ = direct effect of mediator1 on mediator2; b^1^–b^6^ = direct effects of mediators on dependent variables; a^2^b^1^, a^2^b^2^, a^2^b^3^ = indirect effects of predictor on dependent variables through fatigue; a^1^b^4^, a^1^b^5^, a^1^b^6^ = indirect effects of predictor on dependent variables through insomnia; a^1^d^1^b^1^, a^1^d^1^b^2^, a^1^d^1^b^3^ = indirect effect of predictor on dependent variables through insomnia and fatigue in sequence. Dotted line = nonsignificant direct effects, indicating full mediation. All regression coefficients are standardized. * *p* < 0.01, ** *p* < 0.001, ☨ 95% confidence intervals.

The parallel and serial mediating roles of insomnia and fatigue in the relationship between trauma exposure and burnout is visually presented in Figure 1.

Figure 1 encapsulates the obtained results reported in Table 3 and visually shows that the effect of trauma exposure on emotional exhaustion and depersonalization was fully mediated by insomnia and fatigue in sequence. The dotted line indicates that this was full mediation as the relationship between trauma exposure, on the one hand, and emotional exhaustion and depersonalization, on the other hand was nonsignificant in the presence of the two mediators. In addition, Figure 1 also shows that in addition to the serial mediating role of insomnia and fatigue, these two mediators also operated as parallel mediators. In this regard, insomnia mediated the relationships between trauma exposure and all three components of burnout, while fatigue only mediated the relationships between trauma exposure and emotional exhaustion as well as depersonalization.

## 4 Discussion

First responders are disproportionately vulnerable to the development of insomnia, fatigue, and burnout, due to chronic exposure to high‐intensity stressors and potentially traumatic events inherent in their occupational roles [35, 36]. This study investigated the mediating roles of insomnia and fatigue in the relationship between trauma exposure and burnout among South African first responders. There were several significant findings.

First, trauma exposure was associated with increased emotional exhaustion and depersonalization, but not with personal accomplishment. This suggests that frequent exposure to trauma depletes emotional resources and fosters interpersonal detachment without necessarily diminishing perceptions of professional competence or achievement. It is possible that first responders and other trauma‐exposed workers derive a strong sense of purpose from their roles, which buffers against declines in perceived personal accomplishment despite experiencing significant emotional strain. This aligns with previous research suggesting that different dimensions of burnout may be differentially impacted by trauma exposure. For instance, in a study of healthcare professionals, Ilias et al. [37] reported that emotional exhaustion predicted PTSD status, whereas depersonalization and personal accomplishment were not salient predictors. The authors noted that despite exposure to significant stressors and potentially traumatic events, healthcare workers were able to perform their roles competently and did not experience helplessness, suggesting that their sense of self‐efficacy remained intact. Similarly, Stout et al. [38] found that, despite significant trauma exposure, firefighters experienced high emotional exhaustion and moderate depersonalization but did not experience a decline in their sense of personal efficacy or accomplishment. Thus, the findings of the current study are consistent with previous studies suggesting that a strong sense of role purpose and self‐efficacy may buffer against declines in perceived personal accomplishment despite elevated stress [37, 38].

Second, trauma exposure was associated with heightened insomnia and fatigue. In addition, higher levels of insomnia and fatigue, respectively, were associated with higher levels of burnout. One explanation may be related to fear of sleep. Research on trauma‐exposed populations suggests that individuals who have experienced traumatic events often develop anticipatory anxiety around sleep due to the possibility of distressing nightmares and intrusive memories [11, 13, 39]. Fear of sleep contributes to difficulties initiating and maintaining sleep, leading to chronic sleep disturbances and exacerbating physical and emotional exhaustion. As sleep becomes increasingly fragmented and non‐restorative, individuals experience persistent fatigue, which can further impair their psychological resilience and ability to cope with occupational demands and exacerbate symptoms of burnout [11, 35, 40]

Third, insomnia fully mediated the relationship between trauma exposure and the burnout dimensions of emotional exhaustion, depersonalization, and personal accomplishment. This suggests that it is the disruption of sleep that may act as the critical pathway through which trauma exerts its influence on these aspects of burnout. Poor sleep quality likely amplifies emotional depletion and undermines individuals’ capacity to maintain a sense of professional efficacy [41, 42].

Fourth, while fatigue fully mediated the relationships between trauma exposure and emotional exhaustion and depersonalization, it did not mediate the relationship with personal accomplishment. This indicates that although physical and cognitive exhaustion diminish emotional engagement and interpersonal connection, perceptions of professional competence remain relatively resilient, potentially bolstered by intrinsic motivation or organizational support.

Finally, sequential mediation analysis indicated that trauma exposure first leads to insomnia, which subsequently exacerbates fatigue, and together these factors increase emotional exhaustion and depersonalization. However, this pathway did not significantly influence personal accomplishment. This underscores the idea that emotional and interpersonal dimensions of burnout are particularly susceptible to sleep‐related pathways, whereas personal accomplishment may be influenced by additional protective factors.

Together, these findings highlight the differential pathways through which sleep‐related problems and trauma exposure contribute to specific dimensions of burnout. They emphasize the importance of addressing sleep disturbances as part of interventions aimed at reducing burnout symptoms among trauma‐exposed populations. Cognitive‐behavioral therapy for insomnia (CBT‐I) is recognized as the first line treatment for sleep disturbances [43, 44]. CBT‐I targets the maladaptive thoughts and behaviors that sustain insomnia and has demonstrated robust efficacy in improving sleep outcomes. Furthermore, the therapeutic benefits of CBT‐I have been found to extend beyond insomnia to reduce non‐sleep related mental and physical health problems [45]. Thus, integrating CBT‐I into intervention strategies for first responders may improve sleep quality and buffer against the development of burnout and enhance overall psychological resilience.

The study has several limitations that should be acknowledged. First, the sample was restricted to two categories of first responders and was drawn from a specific geographic region, which may limit the generalizability of the findings to other populations or settings. Second, the reliance on self‐report measures introduces the possibility of response biases, such as social desirability or recall bias, which may have influenced participants’ reporting of trauma exposure, sleep disturbances, and burnout symptoms. Finally, while the study employed a quantitative design, future research incorporating qualitative methodologies could provide deeper insight into the lived experiences of trauma‐exposed first responders and offer a more nuanced understanding of the mechanisms linking trauma exposure, sleep disturbances, and burnout.

## 5 Conclusion

This study highlights the critical roles of insomnia and fatigue in mediating the relationship between trauma exposure and burnout among South African first responders. Findings demonstrate that emotional exhaustion and depersonalization are significantly influenced by trauma‐related sleep disturbances and persistent fatigue, while perceptions of personal accomplishment remain comparatively resilient.

These results underscore the importance of early identification and targeted intervention for sleep‐related problems in trauma‐exposed occupational groups. Incorporating evidence‐based treatments such as Cognitive‐Behavioral Therapy for Insomnia (CBT‐I) into first responder mental health programs may be essential to reduce emotional exhaustion, prevent depersonalization, and foster psychological resilience. Prioritizing sleep health could serve as a crucial step in protecting the well‐being of individuals who are routinely exposed to occupational trauma.

## Author Contributions

**Anita Padmanabhanunni:** conceptualization, investigation, writing – original draft, writing – review and editing, project administration, supervision. **Tyrone B. Pretorius:** conceptualization, investigation, writing – original draft, writing – review and editing, methodology, formal analysis.

## Conflicts of Interest

The authors declare no conflicts of interest.

## Transparency Statement

The lead author Tyrone B. Pretorius affirms that this manuscript is an honest, accurate, and transparent account of the study being reported; that no important aspects of the study have been omitted; and that any discrepancies from the study as planned (and, if relevant, registered) have been explained.

## Data Availability

The data that support the findings of this study are available from the corresponding author upon reasonable request.
